# Rainbow connections of bioriented graphs

**DOI:** 10.1016/j.heliyon.2024.e31426

**Published:** 2024-05-21

**Authors:** Linlin Wang, Sujuan Liu, Han Jiang

**Affiliations:** a School of Mathematics, China University of Mining and Technology, Xuzhou, 221116, China; b College of Artificial Intelligence, Tianjin University of Science & Technology, Tianjin, 300457, China

**Keywords:** Bioriented graph, Rainbow connection number, Total rainbow connection number

## Abstract

For a directed graph *D*, it's deemed rainbow connected if each arc is assigned a different color, so that all paths from the vertex *u* to the vertex *v* are rainbow connected. Rainbow connection number refers to how many colors are needed in *D* to achieve rainbow connectivity. Among ordered vertex pair (u,v) with arc coloring, if both arcs and internal vertices exhibit unique colors, it is referred to as a total colored graph. As the total rainbow connection number indicates, the smallest number of colors are required to total-color *D*. This paper focuses on investigating the rainbow connection number of the biorientation of a connected graph, as well as the total rainbow connection number.

## 1 Introduction

We consider the graphs in our paper to be simple and finite, as well as digraphs. It means that the size of any graph is finite and multiple edges, loops in graphs, and multiple arcs in digraphs are not permitted. We will introduce some necessary definitions and notations, for which not defined here are referred to [1], [2].

When all edges in a graph are colored, it is termed as edge-colored. Paths in such graphs are termed *rainbow* paths if there are distinct colors on their edges. A graph *G* with a rainbow path connecting any vertex pair can be called *rainbow connected*. A fascinating challenge emerges when attempting to reduce the number of colors necessary to guarantee rainbow connectivity within graph *G*, with the rainbow connection number, rc(G), representing the minimum amount needed. When *G* is edge-colored, we say a path from *u* to *v* geodesic if it has shortest distance between two vertices and say the graph *G strongly rainbow connected* when it satisfies that any two vertices in *G* can be joined by a rainbow geodesic. To determine the minimum number of colors necessary to ensure strong rainbow connectivity in the graph *G*, we compute the minimum number of colors. The invariant src(G) refers to this minimum number as the strong rainbow connection number for *G*.

First presented in [3], Chartrand et al. propose rainbow connections in graphs. Every new concept in graph theory typically arises from theoretical or practical foundations. For example, Zhang et al. ([4], [7], [6], [5], [8], [9]) have analyzed various structures by utilizing graph invariants to compute topological indices for different molecular structures. Rainbow connectivity finds significant applications in network security, attracting the attention of numerous scholars and yielding substantial results for both general graphs and digraphs. Furthermore recently some new results ([11], [10], [12]) which are concerning the rainbow connectivity in some random graph models are obtained.

For some special graph classes, their rainbow connection numbers have been determined in [13], [14], [15], [16], and in [17], [18], [19], [20], rainbow connection numbers were determined for some graphs with some fixed parameters. A rainbow connection number rc(G) can be determined through NP-hard work, and establishing that rc(G) is less than two will be NP-complete, as demonstrated in [21] by Chakraborty et al. The reader can find more information about the rainbow connection of graphs by examining the survey [22] and the book [23].

Several variations concerning the rainbow connection concept have also been explored, such as rainbow vertex-connection which is discussed in [24], and total rainbow connection which is studied in [25]. When a graph *G* is total-colored, we say it *total rainbow connected* (or *strongly total rainbow connected*) if there always exists a path (or geodesic) whose edges and internal vertices differ in color between any two vertices. Specifically, a connected graph *G* achieves total rainbow connectivity when it necessitates trc(G) colors to attain this property. Further more, we consider the smallest number of colors which make a rainbow connected graph is strongly. For convenience, denote this smallest number by strc(G), which is called the strong total rainbow connectivity.

Dorbec et al., in their referenced work [26], broadened the scope of the rainbow connection concept from traditional graphs to directed graphs, also known as digraphs. A *directed path P* in a digraph is defined by a vertex sequence $v_{0},v_{1},\ldots ,v_{t}$, and the ordered pair $\left(v_{i-1},v_{i}\right)$ for 1≤i≤t, then *P* is referred to as a $v_{0}-v_{t}$ path. In a digraph *D*, if there is a directed path to join any ordered pair of vertices *u* and *v* within the vertex set V(D), it's classified as *strongly connected*, or simply strong. When the arcs of a directed path are colored differently, it is termed as *rainbow*. Rainbow connectivity in a digraph *G* is realized when its arcs are colored, ensuring that any ordered vertex pair in *D* are joined by a rainbow directed path. In this paper we consider the smallest number of colors which make a digraph *D* rainbow connected, and denoted this smallest number by $\overset{\to }{rc}(D)$. If all the arcs of digraph *D* are colored and any two ordered vertex pair are joined by a rainbow path (or geodesic), then we say it is strongly rainbow connected. Similarly, we consider the smallest number of colors to make a digraph *G* strongly rainbow connected, and denote the smallest number by $\overset{\to }{src}(D)$, which is called the *strong rainbow connectivity* of *D*. It is evident that the rainbow connection number $\overset{\to }{rc}(D)$ is larger than diam(D) and less than $\overset{\to }{src}(D)$, where diam(D) is the diameter of *D*. If *G* is a strong oriented graph with *n* vertices, then $\overset{\to }{rc}(G)$ is bounded by *n* [26]. The investigation of rainbow connection in certain special digraph classes was introduced in [27], [28], [29], [30].

Also, Lei et al. posed the notion of total rainbow connection concerning digraphs in their research documented as [31]. When both the ordered pairs (arcs) and internal vertices of a total-colored path receive distinct colors, the path is termed as *total-rainbow*. For any two ordered vertex pair of a total-colored digraph *D*, if there always exists a total-rainbow directed path joined them, then *D* is called in this paper total rainbow connected. Similar to the above, we want to find the smallest number of colors which make a digraph *D* total rainbow connected, and for convenience we denote this smallest number by $\overset{\to }{trc}(D)$, which is also called the *total rainbow connectivity*. If a digraph *D* is not only total rainbow connected, but also satisfies the existence of a total rainbow geodesic between any ordered vertex pair, then we call it strong simply. Similarly, we also consider the smallest number of colors to make a digraph *D* strongly total rainbow connected, and denote this smallest number by $\overset{\to }{strc}(D)$, which is also called *strong total rainbow connectivity* of *D*. It is evident that $\overset{\to }{strc}(D)$ is larger than 2diam(D)−1 and less than $\overset{\to }{trc}(D)$. The rainbow connection connectivity of a digraph is monotonic, i.e., let *H* be a strong spanning subgraph of a digraph *D*, then $\overset{\to }{rc}(D)$ is less than $\overset{\to }{rc}(H)$, and total rainbow connection connectivity has the same property, which means $\overset{\to }{trc}(D)$ is also less than $\overset{\to }{trc}(H)$.

In a digraph *D*, we define two ordered vertex pairs (u,v) and (v,u) as *symmetric* if the arc set of *D* contains both arc (u,v) and arc (v,u); otherwise, the ordered vertex pair is considered *asymmetric*. A digraph *D* is termed an oriented graph if each ordered vertex pair in *D* is asymmetric. If we replace each edge of a graph *G* with the symmetric pair of arcs, then we get a *biorientation* $\overset{↔}{G}$ of a given graph *G*. The length of a path (denoted by ℓ(P)) for a general graph is the number of edges, and for a directed path, it is the number of arcs. If $v_{i},v_{j}\in V(P)$, then $v_{i}Pv_{j}$ represents the part of *P* from $v_{i}$ to $v_{j}$. In their work [31], Lei et al. demonstrated that $\overset{\to }{rc}(\overset{↔}{G})$ is less than rc(G) and $\overset{\to }{trc}(\overset{↔}{G})$ is less than trc(G) for a connected graph *G*. They computed the exact rainbow connection connectivity for the biorientations of various graphs and their total rainbow connectivity.

Theorem 1 *[32]* *(a) For an integer* k≥2 *,* $\overset{\to }{rc}(\overset{↔}{P_{k}})=\overset{\to }{src}(\overset{↔}{P_{k}})=k-1$ *;*

*(b) For an integer* k≥4 *,* $\overset{\to }{rc}(\overset{↔}{C_{k}})=\overset{\to }{src}(\overset{↔}{C_{k}})=\lceil k/2\rceil$ *;*

*(c) Let* t≥2 *. If* ${\overset{↔}{K}}_{k_{1},k_{2},\ldots ,k_{t}}$ *is the complete t-partite digraph where* $k_{i}\geq 2$ *for some i, then* $\overset{\to }{rc}({\overset{↔}{K}}_{k_{1},k_{2},\ldots ,k_{t}})=\overset{\to }{src}({\overset{↔}{K}}_{k_{1},k_{2},\ldots ,k_{t}})=2$ *.*

Theorem 2 *[31]* *(a) For an integer* k≥2 *,* $\overset{\to }{trc}(\overset{↔}{P_{k}})=\overset{\to }{strc}(\overset{↔}{P_{k}})=2k-3$ *.*

*(b) For an integer* k≥3 *,* $\overset{\to }{trc}(\overset{↔}{C_{k}})=\overset{\to }{strc}(\overset{↔}{C_{k}})=g(k)$ *, where*

$$g(k)=\left\{ \begin{array}{cc} k-2,\; & if\;\;k=3,5;\\ k-1,\; & if\;\;4,6,7,8,9,10,12;\\ k,\; & if\;\;k=11\;or\;k\geq 13. \end{array}\right.$$

*(c) For an integer* k≥4 *,* $\overset{\to }{trc}(\overset{↔}{W_{k}})=\overset{\to }{strc}(\overset{↔}{W_{k}})=3$ *, where the wheel* $W_{k}$ *is the graph constructed by taking the cycle* $C_{k}$ *, and joining a new vertex v to every vertex of* $C_{k}$ *.*

*(d) Let* t≥2 *and let* ${\overset{↔}{K}}_{k_{1},k_{2},\ldots ,k_{t}}$ *be a complete t-partite digraph with* $n_{i}\geq 2$ *for some i. Then* $\overset{\to }{trc}({\overset{↔}{K}}_{k_{1},k_{2},\ldots ,k_{t}})=\overset{\to }{strc}({\overset{↔}{K}}_{k_{1},k_{2},\ldots ,k_{t}})=3$ *.*

A fundamental task is to compute the precise values of $\overset{\to }{rc}(\overset{↔}{G})$ and $\overset{\to }{src}(\overset{↔}{G})$ for the biorientation $\overset{↔}{G}$ of a connected graph *G*. This study investigates the above two invariants of the biorientations for two classes of graphs: trees and graphs with diameter 2. The precise values of $\overset{\to }{rc}(\overset{↔}{T}),\overset{\to }{src}(\overset{↔}{T}),\overset{\to }{trc}(\overset{↔}{T})$ and $\overset{\to }{strc}(\overset{↔}{T})$ for a tree *T* are determined, while for a graph *G* with diameter 2, the bounds of $\overset{\to }{rc}(\overset{↔}{G})$ and $\overset{\to }{trc}(\overset{↔}{G})$ are established.

## 2 Main results

First, we give a simple observation. For a connected graph *G*, $diam(G)=diam(\overset{↔}{G})\leq \overset{\to }{rc}(\overset{↔}{G})\leq rc(G)$ and $2diam(G)-1=2diam(\overset{↔}{G})-1\leq \overset{\to }{trc}(\overset{↔}{G})\leq trc(G)$. Hence, we list the simple observation as follows.

Observation 1 Let *G* be a connected graph. We have the following results:

(1) If rc(G)=diam(G), then $\overset{\to }{rc}(\overset{↔}{G})=diam(G)$, but the converse is not true.

(2) If trc(G)=2diam(G)−1, then $\overset{\to }{trc}(\overset{↔}{G})=2diam(G)-1$, but the converse is not true.

The star graph $K_{1,n}$ with n+1 vertices is a counterexample of Observation 1. It is a common understanding that when *n* is an integer greater than or equal to 2, $rc(K_{1,n})=n$, $trc(K_{1,n})=n+1$, $\overset{\to }{rc}({\overset{↔}{K}}_{1,n})=2$ and $\overset{\to }{trc}({\overset{↔}{K}}_{1,n})=3$. If n≥3, then $\overset{\to }{rc}({\overset{↔}{K}}_{1,n})=diam(K_{1,n})<rc(K_{1,n})$ and $\overset{\to }{trc}({\overset{↔}{K}}_{1,n})=2diam(K_{1,n})-1<trc(K_{1,n})$. Therefore, we propose the following problems.

Problem 1 (1) Characterize the graph *G* satisfying that $\overset{\to }{rc}(\overset{↔}{G})=diam(G)$.

(2) Characterize the graph *G* satisfying that $\overset{\to }{trc}(\overset{↔}{G})=2diam(G)-1$.

To delve into the rainbow connection of bioriented trees, we commence by establishing a decomposition method for trees. Let *T* be a tree and L(T) and I(T) represent the set of leaf vertices and inner vertices of *T*, respectively. Notice that V(T)=L(T)∪I(T). We introduce a nest sequence of trees which are subgraphs of *T*. Let $T_{0}=v_{0}v_{1}\ldots v_{d}$ be a longest path in *T*. If $T\neq T_{0}$, then choose a longest path $P_{0}=v_{0,0}v_{0,1}\ldots v_{0,d_{0}}(d_{0}\geq 1)$ in *T* satisfying that $V(T_{0})\cap V(P_{0})=\{v_{0,d_{0}}\}$ and let $T_{1}=T_{0}\cup P_{0}$. If $T\neq T_{1}$, then continue the above procedures. After finite steps, we obtain a nest sequence $T_{0},T_{1},\ldots ,T_{s}$ which is called a *path decomposition* of *T*, satisfying the conditions: (1) $T_{0}$ is a longest path in *T*; (2) for 0≤j<s, $T_{j+1}=T_{j}\cup P_{j}$, where $P_{j}=v_{j,0}v_{j,1}\ldots v_{j,d_{j}}(d_{j}\geq 1)$ is a longest path with $V(T_{j})\cap V(P_{j})=\{v_{j,d_{j}}\}$; (3) $T=T_{s}$. Notice that $\ell (T_{0})=diam(T)$, $\frac{1}{2}\ell (T_{0})\geq \ell (P_{0})\geq \ell (P_{1})\geq \ldots \geq \ell (P_{s-1})$, $L(T)=\{v_{0},v_{d},v_{0,0},v_{1,0},\ldots ,v_{s-1,0}\}$ and $\left\{v_{0,d_{0}},v_{1,d_{1}},\ldots ,v_{s-1,d_{s-1}}\}\subseteq I(T\right)$.

For a tree *T* and two vertices u,v∈V(T), *uTv* represents the only path from *u* to *v* on *T* and $u\overset{↔}{T}\;v$ represents the only directed path from *u* to *v* on $\overset{↔}{T}$. If *c* is an arc-coloring (total-coloring) of $\overset{↔}{T}$, then $c(u\overset{↔}{T}\;v)$ denotes the set of colors appeared on the arcs (arcs and internal vertices) of $u\overset{↔}{T}\;v$.

Theorem 3 *Let T be a tree with* n≥2 *vertices and l leaf vertices. Then* $\overset{\to }{rc}(\overset{↔}{T})=\overset{\to }{src}(\overset{↔}{T})=diam(T)$ *and* $\overset{\to }{trc}(\overset{↔}{T})=\overset{\to }{strc}(\overset{↔}{T})=diam(T)+n-l$ *.*

Proof Since for any two vertices u,v∈V(T), there is exactly one directed path from *u* to *v* on $\overset{↔}{T}$, we obtain that $\overset{\to }{rc}(\overset{↔}{T})=\overset{\to }{src}(\overset{↔}{T})$ and $\overset{\to }{trc}(\overset{↔}{T})=\overset{\to }{strc}(\overset{↔}{T})$. If $T=P_{n}$, the results hold from Theorem 1, Theorem 2. Suppose that $T\neq P_{n}$. Let $T_{0},T_{1},\ldots ,T_{s}(s\geq 1)$ be a path decomposition of *T*, where $T_{0}=v_{0}v_{1}\ldots v_{d}$, $P_{j}=v_{j,0}v_{j,1}\ldots v_{j,d_{j}}(0\leq j<s)$ and $V(P_{j})\cap V(T_{j})=\{v_{j,d_{j}}\}$. Define a map e:V(T)→{0,1,…,d} as follows: for 0≤i≤d, $e(v_{i})=\min\{\ell (v_{i}Tv_{0}),\ell (v_{i}Tv_{d})\}=\min\{i,d-i\}$ and for $v_{j,i}\in V(P_{j})\setminus \{v_{j,d_{j}}\}(0\leq j<s)$, $e(v_{j,i})=\ell (v_{j,i}Tv_{j,0})=i$.

Claim *For a vertex* v∈V(T) *and* $v_{j,d_{j}}(0\leq j<s)$ *,* $e(v)\leq \frac{1}{2}d$ *and* $\ell (P_{j})\leq e(v_{j,d_{j}})$ *.*

Proof of the Claim From the definition, $e(v_{i})\leq \frac{1}{2}d$ for 0≤i≤d. As $T_{0}$ is a longest path in *T*, $\ell (P_{0})\leq \frac{1}{2}d$. Since $\ell (P_{0})\geq \ell (P_{1})\geq \ldots \geq \ell (P_{s-1})$ and $e(v_{j,i})\leq \ell (P_{j})$ for $0\leq i<d_{j}$, we have $e(v_{j,i})\leq \frac{1}{2}d$. Hence, for any vertex v∈V(T), $e(v)\leq \frac{1}{2}d$. Since $T_{0}$ is a longest path in *T* and every $P_{j}$ is a longest path added to $T_{j}$, we have $\ell (P_{j})\leq e(v_{j,d_{j}})$, so the Claim is proved.

**Figure 1:**
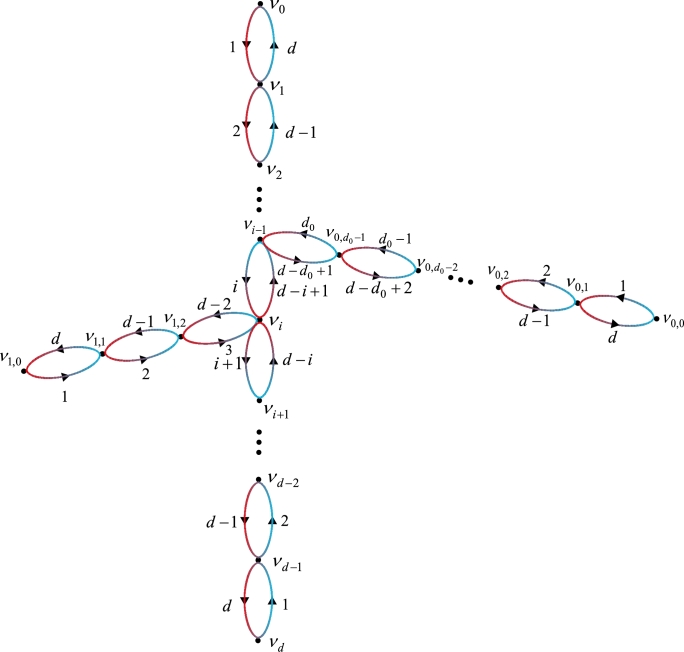
The arc-coloring *c* of a bioriented tree.

(1) In order to prove $\overset{\to }{rc}(\overset{↔}{T})\leq d$, define an arc-colorings *c* of $\overset{↔}{T}$ as follows: for 1≤i≤d, $c(v_{i-1}v_{i})=i$ and $c(v_{i}v_{i-1})=d-i+1$; for 0≤j<s and $1\leq i\leq d_{j}$, $c(v_{j,i-1}v_{j,i})=i$ and $c(v_{j,i}v_{j,i-1})=d-i+1$. Notice that *c* uses *d* (i.e., diam(T)) colors. Fig. 1 shows the arc-coloring *c* of a bioriented tree $\overset{↔}{T}$. By induction, we will show that $\overset{↔}{T_{i}}(0\leq i\leq s)$ satisfies two conditions:

(C1) $\overset{↔}{T_{i}}$ is rainbow connected;

(C2) For any vertices $u,v\in V(T_{i})$, $c(u\overset{↔}{T}\;v)\subseteq \{e(u)+1,e(u)+2,\ldots ,d\}$ and $c(u\overset{↔}{T}\;v)\subseteq \{1,2,\ldots ,d-e(v)\}$.

Obviously, $\overset{↔}{T_{0}}$ is rainbow connected. Let $u,v\in V(T_{0})$. Suppose that $u=v_{i}$ and $v=v_{j}$. Since $c(u\overset{↔}{T}\;v_{0})=\{d-i+1,d-i+2,\ldots ,d\}\subseteq \{e(u)+1,e(u)+2,\ldots ,d\}$, $c(u\overset{↔}{T}\;v_{d})=\{i+1,i+2,\ldots ,d\}\subseteq \{e(u)+1,e(u)+2,\ldots ,d\}$ and $u\overset{↔}{T}\;v$ is a subgraph of $u\overset{↔}{T}\;v_{0}$ or $u\overset{↔}{T}\;v_{d}$, we obtain that $c(u\overset{↔}{T}\;v)\subseteq \{e(u)+1,e(u)+2,\ldots ,d\}$. Similarly, we can derive $c(u\overset{↔}{T}\;v)\subseteq \{1,2,\ldots ,d-e(v)\}$ from $c(v_{0}\overset{↔}{T}\;v)=\{1,2,\ldots ,j\}\subseteq \{1,2,\ldots ,d-e(v)\}$ and $c(v_{d}\overset{↔}{T}\;v)=\{1,2,\ldots ,d-j\}\subseteq \{1,2,\ldots ,d-e(v)\}$. Hence, $\overset{↔}{T_{0}}$ satisfies conditions (C1) and (C2).

Assume that $\overset{↔}{T_{j}}(0\leq j<s)$ satisfies conditions (C1) and (C2). Now consider ${\overset{↔}{T}}_{j+1}=\overset{↔}{T_{j}}\cup \overset{↔}{P_{j}}$. Let $u,v\in V(T_{j+1})$. If $u,v\in V(T_{j})$ or $u,v\in V(P_{j})$, then $u\overset{↔}{T}\;v$ and $v\overset{↔}{T}\;u$ are rainbow paths from the assumption and the definition of *c*. Suppose that $u\in V(T_{j})\setminus \{v_{j,d_{j}}\}$ and $v\in V(P_{j})\setminus \{v_{j,d_{j}}\}$. Since $c(u\overset{↔}{T}\;v_{j,d_{j}})\subseteq \{1,2,\ldots ,d-e(v_{j,d_{j}})\}$, $c(v_{j,d_{j}}\overset{↔}{T}\;v_{j,0})=\{d,d-1,\ldots ,d-\ell (P_{j})+1\}$ and $\ell (P_{j})\leq e(v_{j,d_{j}})$, we have $c(u\overset{↔}{T}\;v_{j,d_{j}})\cap c(v_{j,d_{j}}\overset{↔}{T}\;v_{j,0})=\emptyset$, implying that $u\overset{↔}{T}\;v_{j,0}$ is a rainbow path. Similarly, $v_{j,0}\overset{↔}{T}\;u$ is a rainbow path from $c(v_{j,0}\overset{↔}{T}\;v_{j,d_{j}})=\{1,2,\ldots ,\ell (P_{j})\}$ and $c(v_{j,d_{j}}\overset{↔}{T}\;u)\subseteq \{e(v_{j,d_{j}})+1,e(v_{j,d_{j}})+2,\ldots ,d\}$. As $u\overset{↔}{T}\;v$ and $v\overset{↔}{T}\;u$ are subgraphs of $u\overset{↔}{T}\;v_{j,0}$ and $v_{j,0}\overset{↔}{T}\;u$, respectively, $u\overset{↔}{T}\;v$ and $v\overset{↔}{T}\;u$ are rainbow paths. Hence, ${\overset{↔}{T}}_{j+1}$ is rainbow connected.

Let u,v be vertices of $T_{j+1}$. We distinguish four cases to show that ${\overset{↔}{T}}_{j+1}$ satisfies condition (C2).

Case 1. $u,v\in V(T_{j})$.

In this case, the condition (C2) holds from the assumption.

Case 2. $u,v\in V(P_{j})$.

From the definition of *c* and the above claim, we get that $c(u\overset{↔}{T}\;v_{j,0})=\{d-e(u)+1,d-e(u)+2,\ldots ,d\}\subseteq \{e(u)+1,e(u)+2,\ldots ,d\}$ and $c(u\overset{↔}{T}\;v_{j,d_{j}})=\{e(u)+1,e(u)+2,\ldots ,\ell (P_{j})\}\subseteq \{e(u)+1,e(u)+2,\ldots ,d\}$. Since $u\overset{↔}{T}\;v$ is a subgraph of $u\overset{↔}{T}\;v_{j,0}$ or $u\overset{↔}{T}\;v_{j,d_{j}}$, we have $c(u\overset{↔}{T}\;v)\subseteq \{e(u)+1,e(u)+2,\ldots ,d\}$. Similarly, $c(u\overset{↔}{T}\;v)\subseteq \{1,2,\ldots ,d-e(v)\}$ from $c(v_{j,0}\overset{↔}{T}\;v)=\{1,2,\ldots ,e(v)\}$ and $c(v_{j,d_{j}}\overset{↔}{T}\;v)=\{d-e(v),d-e(v)-1,\ldots ,d-\ell (P_{j})+1\}$.

Case 3. $u\in V(T_{j})\setminus \{v_{j,d_{j}}\}$ and $v\in V(P_{j})\setminus \{v_{j,d_{j}}\}$.

From the assumption and the definition of *c*, we have $c(u\overset{↔}{T}\;v_{j,d_{j}})\subseteq \{e(u)+1,e(u)+2,\ldots ,d\}$ and $c(v_{j,d_{j}}\overset{↔}{T}\;v_{j,0})=\{d,d-1,\ldots ,d-\ell (P_{j})+1\}$. Since $\ell (P_{j})\leq e(v_{j,d_{j}})\leq \frac{1}{2}d$ and $u\overset{↔}{T}\;v_{j,0}=(u\overset{↔}{T}\;v_{j,d_{j}})\cup (v_{j,d_{j}}\overset{↔}{T}\;v_{j,0})$, we obtain that $c(u\overset{↔}{T}\;v_{j,0})\subseteq \{e(u)+1,e(u)+2,\ldots ,d\}$. As $u\overset{↔}{T}\;v\subseteq u\overset{↔}{T}\;v_{j,0}$, $c(u\overset{↔}{T}\;v)\subseteq \{e(u)+1,e(u)+2,\ldots ,d\}$. Similarly, $c(u\overset{↔}{T}\;v)\subseteq \{1,2,\ldots ,d-e(v)\}$ from $c(u\overset{↔}{T}\;v_{j,d_{j}})\subseteq \{1,2,\ldots ,d-e(v_{j,d_{j}})\}$ and $c(v_{j,d_{j}}\overset{↔}{T}\;v)=\{d-e(v),d-e(v)-1,\ldots ,d-\ell (P_{j})+1\}$.

Case 4. $u\in V(P_{j})\setminus \{v_{j,d_{j}}\}$ and $v\in V(T_{j})\setminus \{v_{j,d_{j}}\}$.

In this case, it can be proved that (C2) is satisfied similar to case 3.

Therefore, ${\overset{↔}{T}}_{j+1}$ satisfies conditions (C1) and (C2). We conclude that *c* is a rainbow arc-coloring of $\overset{↔}{T}$, i.e., $\overset{\to }{rc}(\overset{↔}{T})\leq diam(T)$. As $\overset{\to }{rc}(\overset{↔}{T})\geq diam(T)$ obviously, we have $\overset{\to }{rc}(\overset{↔}{T})=diam(T)$.

(2) In order to show $\overset{\to }{trc}(\overset{↔}{T})\leq diam(T)+n-l$, a total-coloring $c^{\prime }$ of $\overset{↔}{T}$ is defined as follows: for an arc $a\in A(\overset{↔}{T})$, $c^{\prime }(a)=c(a)$, color the n−l inner vertices of $\overset{↔}{T}$ with distinct n−l new colors and color the leaf vertices of $\overset{↔}{T}$ with a color of *c*. Since *c* is a rainbow arc-coloring of $\overset{↔}{T}$ and the inner vertices are colored with distinct new colors, every directed path of $\overset{↔}{T}$ is a total rainbow path under the total-coloring $c^{\prime }$. Hence, $\overset{↔}{T}$ is total rainbow connected, implying that $\overset{\to }{trc}(\overset{↔}{T})\leq diam(T)+n-l$.

In a total rainbow coloring of $\overset{↔}{T}$, the arc set $A(\overset{↔}{T})$ needs at least diam(T) colors, obviously. As any two inner vertices must be the internal vertices of a directed path between two leaf vertices of $\overset{↔}{T}$, the inner vertices need n−l distinct colors in a total rainbow coloring of $\overset{↔}{T}$. Since any inner vertex and an arc of $\overset{↔}{T}$ must appear on a directed path between two leaf vertices, we obtain that $c(I(T))\cap c(A(\overset{↔}{T}))=\emptyset$ in a total rainbow coloring. Therefore, $\overset{\to }{trc}(\overset{↔}{T})\geq diam(T)+n-l$. We conclude that $\overset{\to }{trc}(\overset{↔}{T})=diam(T)+n-l$. □

A widely acknowledged fact is that a connected graph *G* with radius *r* possesses a spanning tree with a diameter of at most 2*r*. Leveraging Theorem 3 alongside the monotonicity principle of the rainbow connection number in digraphs, we derive the subsequent results.

Corollary 1 *Let G be a connected graph and T a spanning tree with minimum diameter among all spanning trees of G. We have* $\overset{\to }{rc}(\overset{↔}{G})\leq diam(T)\leq 2rad(G)$ *, where* rad(G) *is the radius of G.*

Corollary 2 *Let t be an integer with* t≥0 *. Then there exists a connected graph G satisfying that* $rc(G)-\overset{\to }{rc}(\overset{↔}{G})\geq t$ *,* $src(G)-\overset{\to }{src}(\overset{↔}{G})\geq t$ *,* $trc(G)-\overset{\to }{trc}(\overset{↔}{G})\geq t$ *and* $strc(G)-\overset{\to }{strc}(\overset{↔}{G})\geq t$ *.*

Assuming *G* is a connected graph with a diameter of 2, we examine the bounds of rainbow connection number and total rainbow connection number of $\overset{↔}{G}$.

Theorem 4 *Let G be a connected graph with diameter 2. Then* $2\leq \overset{\to }{rc}(\overset{↔}{G})\leq 3$ *and the bounds are tight;* $3\leq \overset{\to }{trc}(\overset{↔}{G})\leq 4$ *.*

Proof As diam(G)=2, we have $\overset{\to }{rc}(\overset{↔}{G})\geq 2$ and $\overset{\to }{trc}(\overset{↔}{G})\geq 3$ by Observation 1. Now we start to prove that $\overset{\to }{rc}(\overset{↔}{G})\leq 3$. When Δ(G)=n−1, where Δ(G) and *n* are the maximum degree and order of *G* respectively, then $K_{1,n-1}$ is a spanning subgraph of *G*. Since $2=diam(G)\leq \overset{\to }{rc}(\overset{↔}{G})\leq \overset{\to }{rc}({\overset{↔}{K}}_{1,n-1})=2$, we have $\overset{\to }{rc}(\overset{↔}{G})=2$. Suppose that Δ(G)<n−1. Let *r* be a vertex of *G*, and the set $V_{i}(0\leq i\leq 2)$ consists of all the vertices *v* of *G* with $d_{G}(r,v)=i$. Notice that $V_{0}=\{r\}$, $V_{2}\neq \emptyset$ and $V(G)=V_{0}\cup V_{1}\cup V_{2}$. Define an arc-coloring *c* of $\overset{↔}{G}$ as follows: for an arc $uv\in A(\overset{↔}{G})$, if u=r and $v\in V_{1}$, then c(uv)=1; if $u\in V_{1}$ and v=r, then c(uv)=2; if $u\in V_{1}$ and $v\in V_{2}$, then c(uv)=2; if $u\in V_{2}$ and $v\in V_{1}$, then c(uv)=1; if $u,v\in V_{1}$ or $u,v\in V_{2}$, then c(uv)=3. It can be checked that *c* is a rainbow arc-coloring of $\overset{↔}{G}$, i.e., $\overset{\to }{rc}(\overset{↔}{G})\leq 3$.

Consider the cycles $C_{4}$ and $C_{5}$ with $diam(C_{4})=diam(C_{5})=2$. From Theorem 1, $\overset{\to }{rc}(\overset{↔}{C_{4}})=2$ and $\overset{\to }{rc}(\overset{↔}{C_{5}})=3$. Hence, the bounds of rainbow connection number of $\overset{\to }{rc}(\overset{↔}{G})$ are tight.

In order to show $\overset{\to }{trc}(\overset{↔}{G})\leq 4$, define a total-coloring $c^{\prime }$ as follows: for an arc $a\in A(\overset{↔}{G})$, $c^{\prime }(a)=c(a)$ and for v∈V(G), $c^{\prime }(v)=4$. It can be checked that $c^{\prime }$ is a total rainbow coloring of $\overset{↔}{G}$ with 4 colors, i.e., $\overset{\to }{trc}(\overset{↔}{G})\leq 4$. □

## 3 Conclusion

This study firstly exam the rainbow connection number and total rainbow connection number of biorientations in two distinct categories of graphs. There are several important questions regarding the rainbow connectivity and total rainbow connectivity of biorientations that require further exploration. One of the relationships studied is the connection between the rainbow connection number and total rainbow connection number with the diameter of biorientations.

## Funding

This paper is supported by 10.13039/501100001809NSFC No. 11401434, 10.13039/501100001809NSFC No. 11501561, 10.13039/501100001809NSFC No. 11971347.

## CRediT authorship contribution statement

**Linlin Wang:** Writing – review & editing, Writing – original draft, Methodology, Funding acquisition. **Sujuan Liu:** Writing – review & editing, Writing – original draft, Methodology, Funding acquisition. **Han Jiang:** Writing – review & editing, Writing – original draft, Methodology.

## Declaration of Competing Interest

The authors declare that they have no known competing financial interests or personal relationships that could have appeared to influence the work reported in this paper.

## Data Availability

No data was used for the research described in the article.
